# Supplementary figures and images for: Context effects on probability estimation
Source: PLoS Biol. 2020 Mar 5;18(3):e3000634. doi: 10.1371/journal.pbio.3000634 (PMC7077880; doi:10.1371/journal.pbio.3000634)

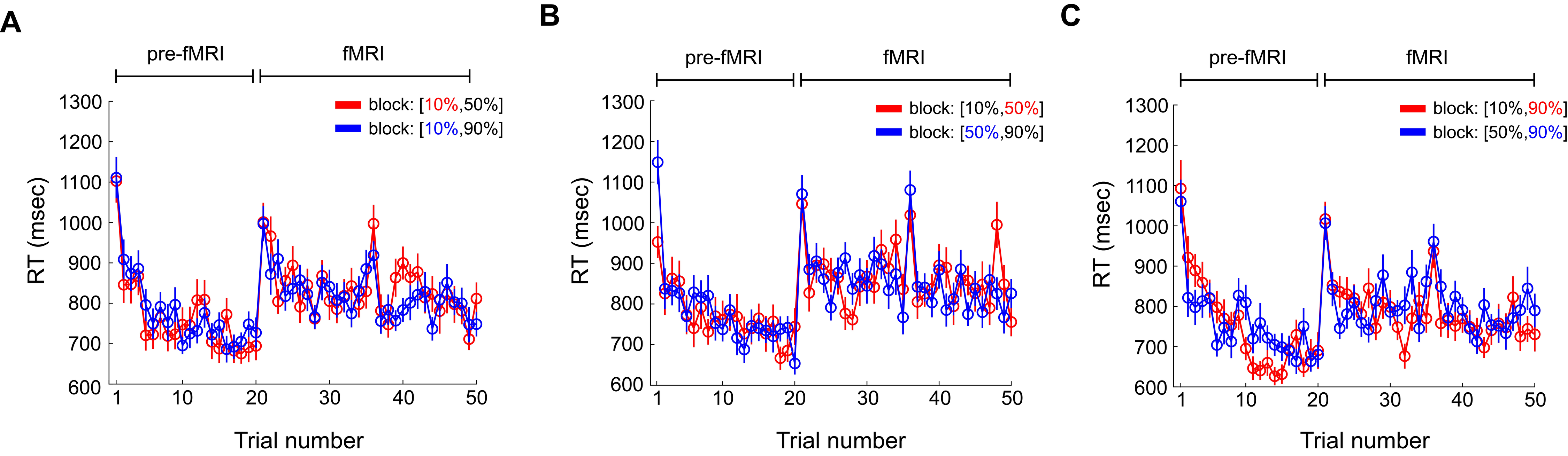

Supplement: S1 Fig — Here, we plot the dynamics of mean RT (across subjects) over the course of the experiment (pre-fMRI session and fMRI session) separately for each reward probability in each context. (A) 10% reward. (B) 50% reward. (C) 90% reward. Conventions are the same as Fig 4. fMRI, functional magnetic resonance imaging; RT, response time (TIF) [file pbio.3000634.s001.tif]

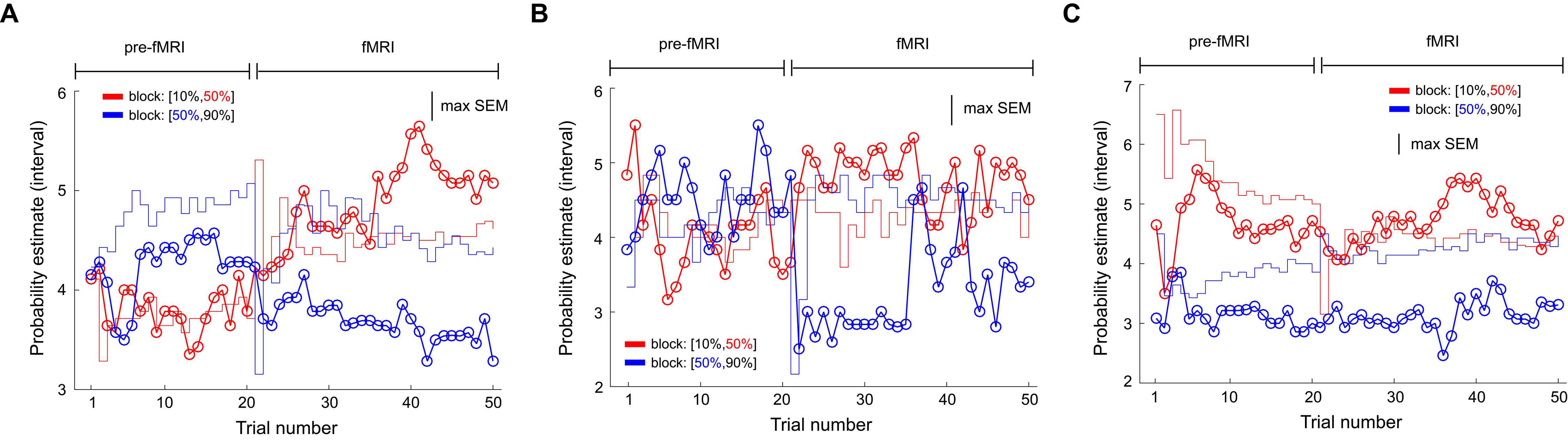

Supplement: S2 Fig — It is possible that context effect on the 50% probability estimates (Fig 4B) was driven by reward frequency bias in the pre-fMRI session, with 50% reward in the [10%, 50%] context (red) having larger reward frequency than that in the [50%, 90%] context (blue). To address this issue, we divided subjects into three subgroups according to their experience in the pre-fMRI session and plotted average probability estimates for each subgroup separately. We found context effect consistent with Fig 4B across all three subgroups. (A) Subgroup 1 (14 subjects): Subjects who experienced smaller reward frequency when facing the 50% reward stimulus in the [10%, 50%] context than the 50% reward stimulus in the [50%, 90%] context in the pre-fMRI session. (B) Subgroup 2 (6 subjects): Subjects who experienced the same reward frequency when facing the 50% reward stimuli between [10%, 50%] and [50%, 90%] contexts. (C) Subgroup 3 (14 subjects): Subjects who experienced larger reward frequency when facing the 50% reward stimulus in the [10%, 50%] context than the 50% reward stimulus in the [50%, 90%] context. We found that the subjects in all three subgroups showed the context effect consistent with Fig 4B. That is, regardless of the subjects’ experience in the pre-fMRI session, for 50% reward, they gave larger probability estimates in the [10%, 50%] context (red) than in the [50%, 90%] context (blue). This suggests that the context effect shown in Fig 4B is not because of bias in reward frequency the subjects experienced in the pre-fMRI session. fMRI, functional magnetic resonance imaging (TIF) [file pbio.3000634.s002.tif]

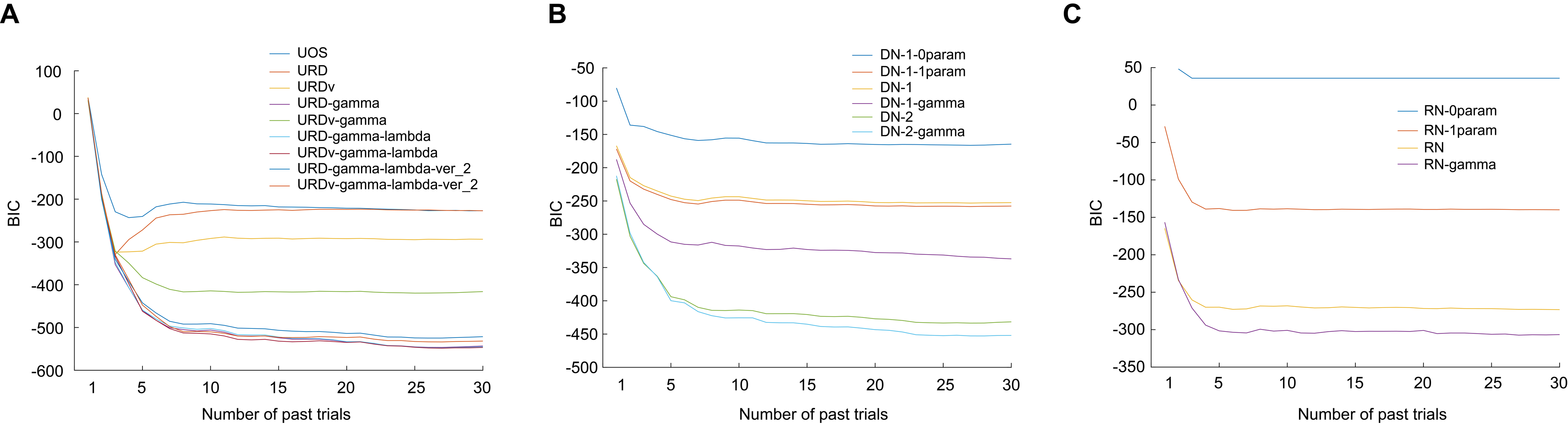

Supplement: S3 Fig — In the paper, we fit different models to subjects’ probability estimates. In one class of models (the non-Rescorla–Wagner model framework), we considered a time window into the past—namely, the number of past trials—when calculating the reward frequency and variance statistics used by the models. In this case, the number of past trials became a free parameter. We found that the model fits—in BIC values—decreased as a function of window length and would vary little after 5 trials into the past. Here, we show BIC values based on fitting group average data plotted against number of past trials considered to compute frequency statistics for model computations. (A) The URD models (9 versions). (B) DN models (6 versions). (C) RN models (4 versions). Model abbreviations are the same as in the main text. In addition, UOS is for a version of URD in which the frequency of overall reward is replaced by the reward frequency of the OS, and URDv is a version of the URD in which the estimated standard deviation of potential outcomes is replaced by the estimated variance of potential outcomes. URD version 2 (ver_2) is a version of URD in which both reward frequency of the stimulus of interest and the overall reward frequency are transformed into probability weight based on the weighting function with free parameter γ (gamma; Eq 6 in “Materials and methods”) when computing the probability estimate. BIC, Bayesian information criterion; DN, divisive normalization; OS, the other stimulus present in the same context as the stimulus of interest; RN, range normalization; URD, uncertainty and reference dependent. (TIF) [file pbio.3000634.s004.tif]

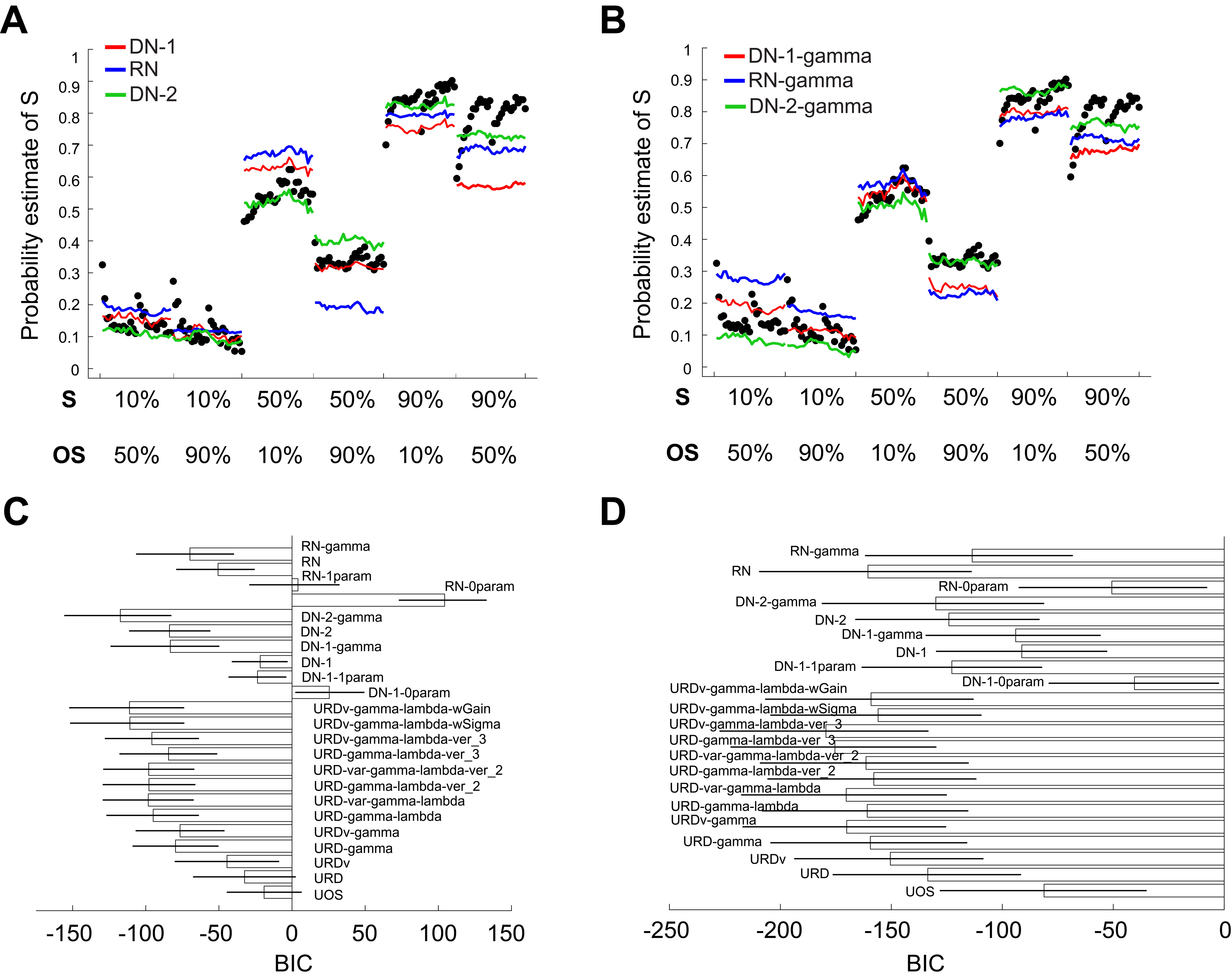

Supplement: S4 Fig — As described in the main text, we fit computational models at both the group and individual-subject levels. When fitting individual-subject data, we considered two model frameworks—the Rescorla–Wagner and the non-Rescorla–Wagner model frameworks. Here, we show model fits from the non-Rescorla–Wagner framework (A–B) and the BICs from both frameworks (C–D). (A–B) Fitting results from the non-Rescorla–Wagner framework. (A) Model fits of DN-1, DN-2, and RN. (B) Model fits of DN-1-γ, DN-2-γ, and RN-γ. (C–D) Model comparison based on BIC. (C) Non-Rescorla–Wagner framework (23 models). (D) Rescorla–Wagner framework (22 models, without RN-1param due to poor convergence). Model abbreviations are the same as in S3 Fig. In addition, version 3 (ver_3) of URD is a version of URD in which loss aversion is performed first before probability weighting, _wSigma indicates a version of URD in which a free weighting parameter is multiplied with estimated uncertainty (either the standard deviation or variance of potential outcomes) in the URD computation, and _wGain is a version of URD in which a free weighting parameter is multiplied with the reference-dependent term σ^S(wp−foverall) in Eq 8 in Materials and methods when wp−foverall>0. BIC, Bayesian information criterion; DN, divisive normalization; RN, range normalization; URD, uncertainty and reference dependent. (TIF) [file pbio.3000634.s005.tif]

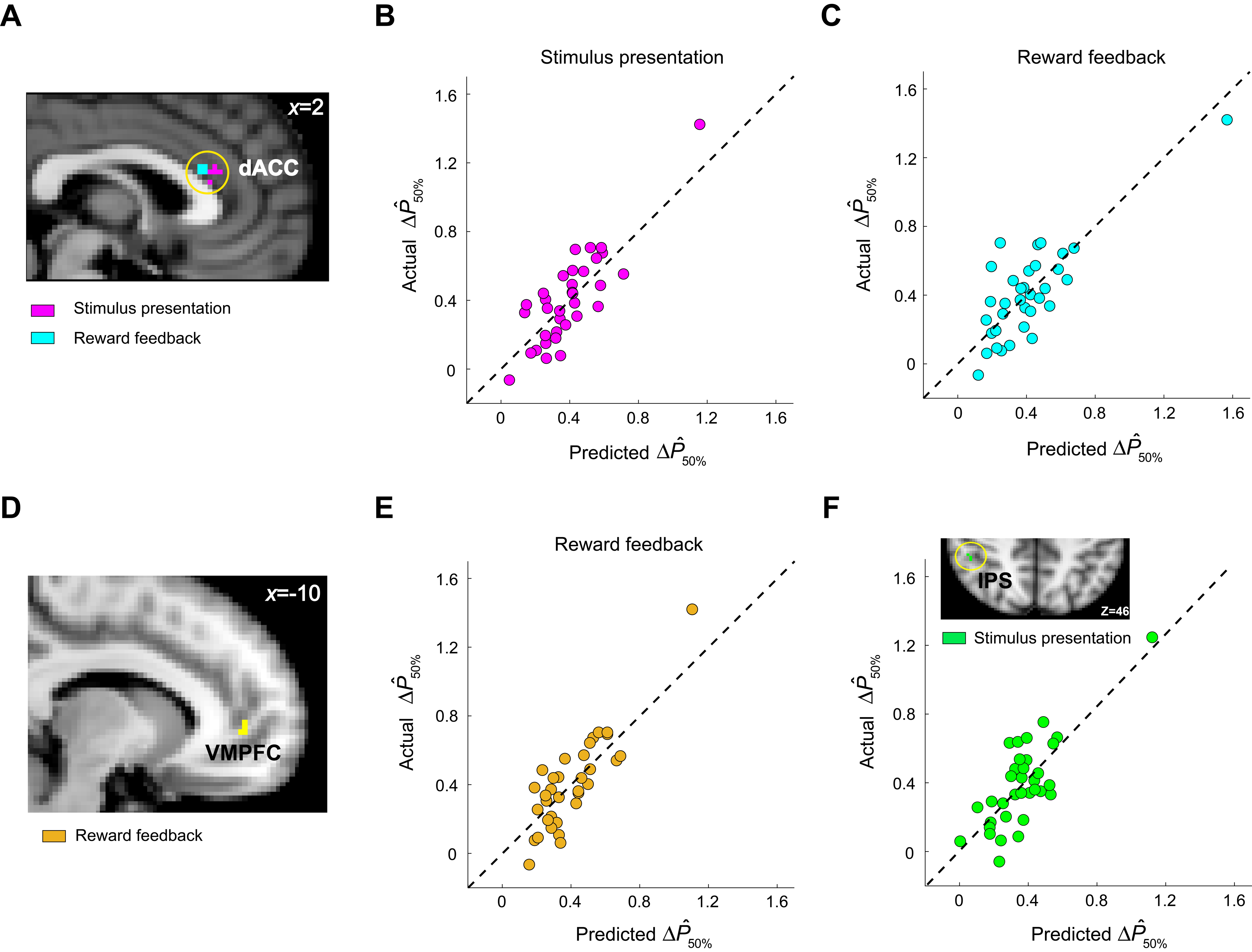

Supplement: S5 Fig — Conventions are the same as in Fig 9 in the main text. In Fig 9, we present MVPA results that excluded one subject’s data because she or he had too many missing trials (she or he did not provide probability estimate within two seconds after stimulus onset in 1/3 of the 50% reward trials), making the estimates of BOLD response less reliable compared with other subjects. Here, we show results from including this subject’s data in the analysis. As expected, the results are not identical to those shown in Fig 9. However, they are similar in the sense that dACC—at both stimulus presentation and reward feedback—represented individual subjects’ context effect on probability estimates (S5A, S5B and S5C Fig), VMPFC represented individual subjects’ context effect on probability estimates at the time of reward feedback (S5D and S5E Fig), and right IPS represented individual subjects’ context effect on probability estimates at the time of stimulus presentation (S5F Fig). BOLD, Blood oxygen level dependent; dACC, dorsal anterior cingulate cortex; IPS, intraparietal sulcus; MVPA, multivoxel pattern analysis; VMPFC, ventromedial prefrontal cortex (TIF) [file pbio.3000634.s006.tif]

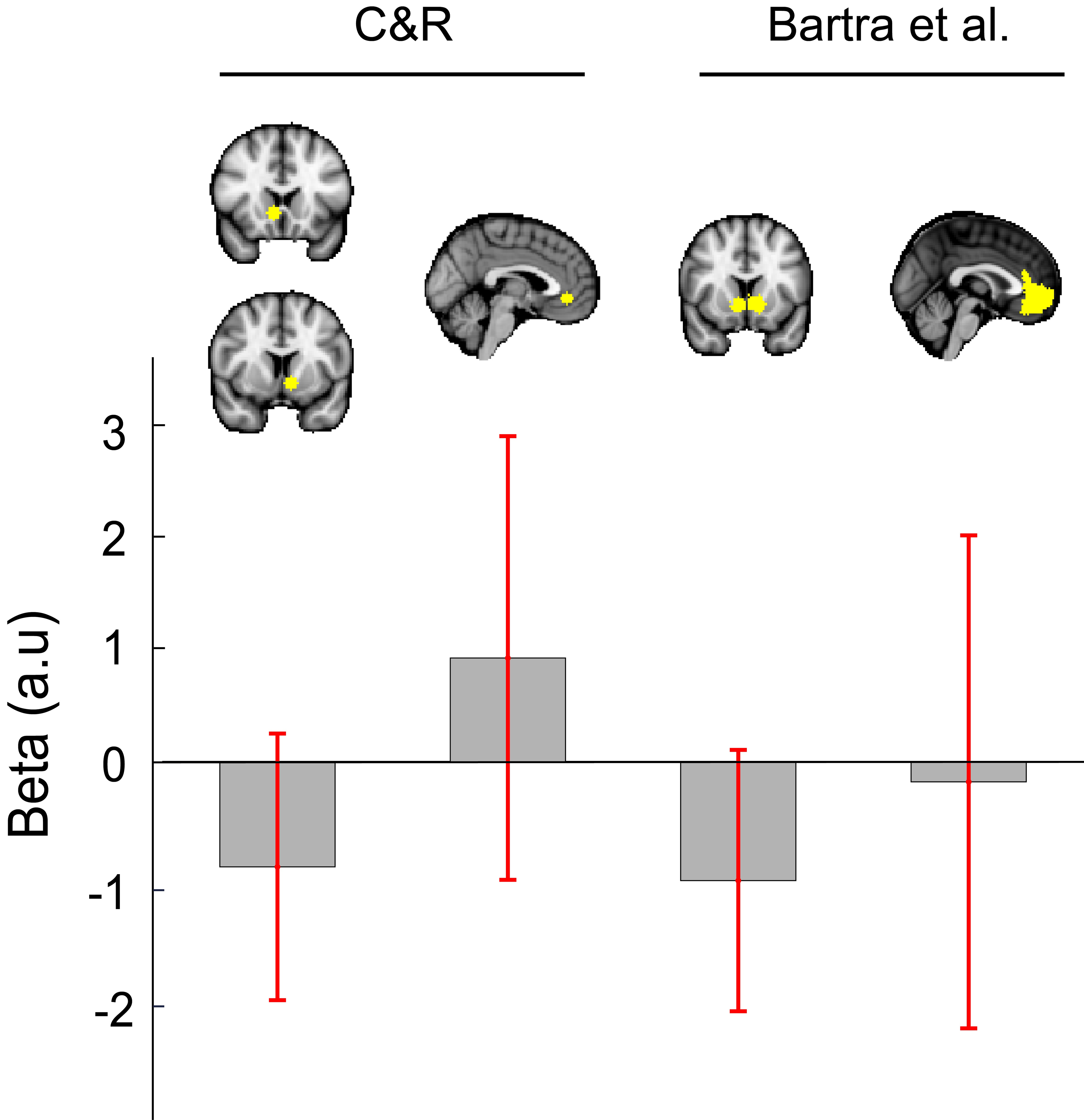

Supplement: S6 Fig — To investigate neural representations for probability estimates, we ran a GLM identical to GLM-1 (see the subsection “General linear modeling of BOLD response” in “Materials and methods”) with the exception of adding a parametric regressor representing subjects’ trial-by-trial probability estimates at the time of stimulus presentation. At the whole-brain level, we did not find regions that significantly correlated with probability estimates. We performed ROI analysis in the VMPFC and VS based on previous meta-analysis papers in value-based decision making and also did not find these ROIs to represent trial-by-trial probability estimates. The ROIs used were identical to those shown in Fig 10 in the main text. Here, we show results from using sphere masks (radius = 8 mm) centered at the peak coordinates for subjective value in VMPFC ([x−2, y40, z−6]) and VS ([x−8, y8, z−6]) identified in Clithero and Rangel. The mean beta value was not significantly different from 0 in both ROIs (VS: t = −0.788, df = 33, p = 0.437; VMPFC: t = 0.468, df = 33, p = 0.643). We also used masks from Bartra and colleagues and did not see the beta value of probability estimate to differ significantly from 0 (VS: t = −0.91, df = 33, p = 0.37; VMPFC: t = −0.08, df = 33, p = 0.936). In summary, we did not find VMPFC and VS to represent subjects’ trial-by-trial probability estimates at the time of stimulus presentation. GLM, general linear model; ROI, region of interest; VMPFC, ventromedial prefrontal cortex; VS, ventral striatum (TIF) [file pbio.3000634.s007.tif]

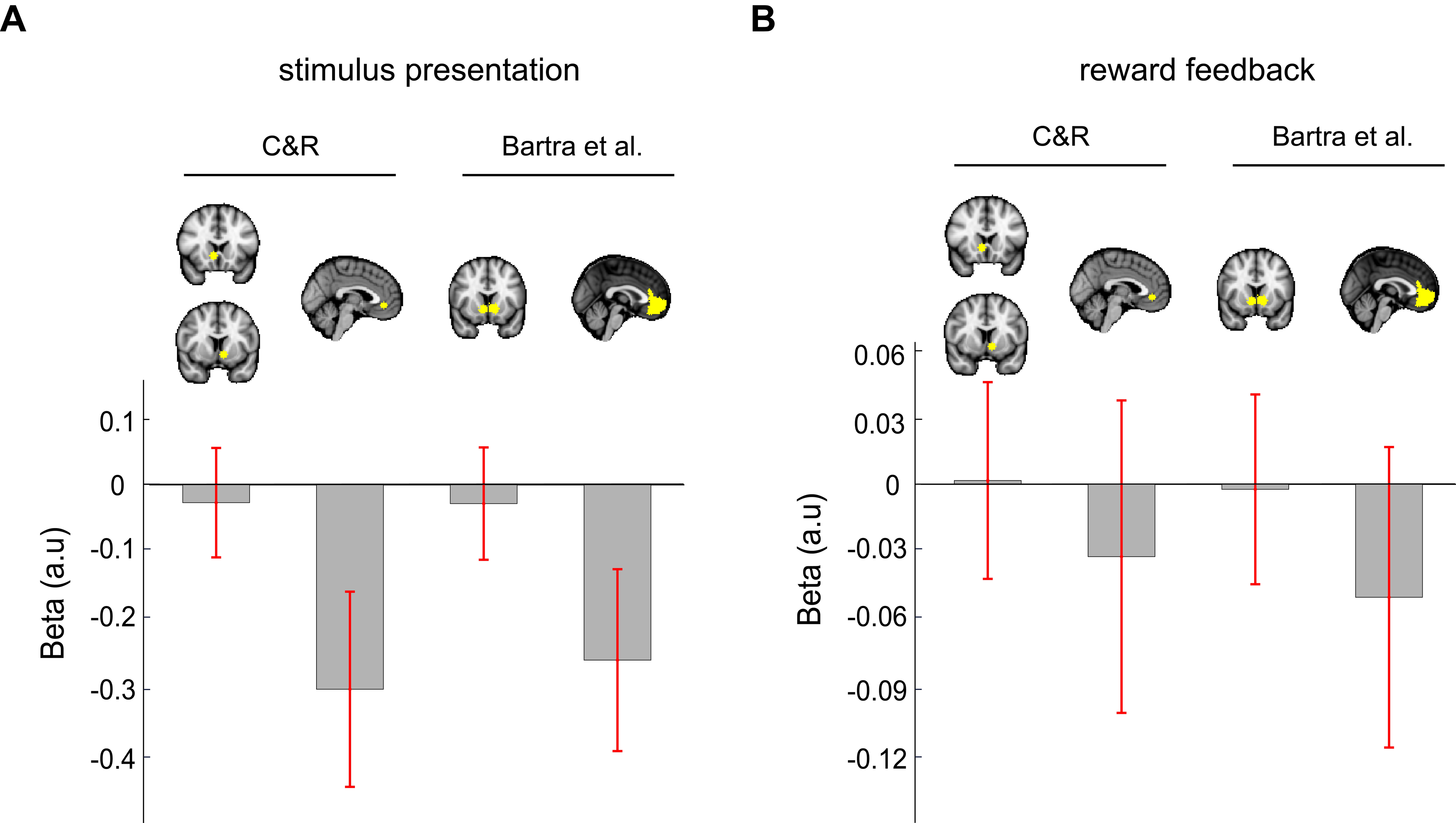

Supplement: S7 Fig — In this study, we found that dACC represented individual differences in context effect on probability estimates based on MVPA analysis (Fig 9). To further examine whether dACC showed task-related functional connectivity with regions shown to represent reward statistics, namely, VS for representing the overall frequency of reward associated with a particular context, we performed the following PPI analysis using dACC as the seed region (sphere mask with 8 mm radius centered at [x2, y30, z18]—the voxel with the strongest effect in MVPA analysis). The PPI model implemented two PPI contrasts, one for the interaction between the dACC time series and the onset regressor at the time of stimulus presentation and the other for the interaction between seed time series and onset regressor at the time of reward feedback. These two contrasts allowed us to examine regions that show changes in functional connectivity with dACC at the time of stimulus presentation and at the time of reward feedback separately. The rest of the regressors in the PPI model were identical to GLM-1 (see the subsection “General linear modeling of BOLD response” in “Materials and methods”). We performed ROI analysis in VMPFC and VS based on previous meta-analysis papers in value-based decision making and found that dACC did not show changes in functional connectivity with VS at both time windows but showed decrease in functional connectivity with VMPFC at the time of stimulus presentation. The ROIs used were identical to those shown in Fig 10. (A) ROI analysis on PPI contrast at the time of stimulus presentation. The beta value represents the regression coefficient of the PPI contrast. (Left two bars) VS and VMPFC ROIs from Clithero and Rangel: VS: t = −0.327, df = 33, p = 0.746; VMPFC: t = −2.094, df = 33, p = 0.044. (Right two bars) VS and VMPFC ROIs from Bartra and colleagues: VS: t = −0.335, df = 33, p = 0.74; VMPFC: t = −1.93, df = 33, p = 0.063. (B) ROI analysis on PPI contrast at the time of reward [file pbio.3000634.s008.tif]
