# Supplementary material for: Context effects on probability estimation
Source: PLoS Biol. 2020 Mar 5;18(3):e3000634. doi: 10.1371/journal.pbio.3000634 (PMC7077880; doi:10.1371/journal.pbio.3000634)
Supplement: S1 Text — Here, we describe in detail how we fit different computational models for probability estimation in the Rescorla–Wagner reinforcement-learning model framework. (DOCX) [file pbio.3000634.s003.docx]

In each experiment we performed, there were three different contexts, each run in separate blocks of trials. For example, in Experiment 1, there were the [10%,50%], [10%,90%] and [50%,90%] contexts.

In the model-fitting exercise, we fit 9 different models and subsequently performed model comparison using Bayesian Information Criterion (BIC) (Fig. 7). In all these models, we assume that subjects retrieve past outcomes – with a free parameter on how many trials they retrieve from the past – to compute reward-frequency and variance statistics and use them to compute estimate on probability of reward. In other words, we did not consider possible memory decay that would lead to decrease in weighting outcomes into the distant past. This feature can be captured by the Rescorla-Wagner reinforcement learning (RL) models – often referred to as model-free RL – that use the delta-learning rule

$V_{t+1}=V_{t}+\delta_{t}$ (1)

where value estimate at time t+1 ($V_{t+1}$) is updated based on value estimate at time t ($V_{t}$) plus prediction error ($\delta_{t}$) that is computed based on the following equation

$\delta_{t}=\alpha\left(R_{t}-V_{t}\right)$ (2)

where $R_{t}$ is the reward outcome at t, $V_{t}$ is the value estimate at t, and $\alpha$ is the free parameter that captures learning rate.

What we then did was to incorporate the Rescorla-Wagner model framework to all the context-dependent models we fit before. When we fit these models, we fit for each context separately using maximum likelihood. That is, for each context separately, the best-fitted parameter estimates in each model we fit maximize the likelihood of the model given the data of that context. This is another important departure from the original model-fitting exercise where we fit the models using all data.

Below, we describe each model we fit. But before describing each model in detail, we remind the readers about our task: in each context (a block of trials), subjects on each trial faced one of two possible visual stimuli. Each stimulus carried a particular probability of reward but the subjects did not know about it. She or he had to learn it through reward outcome feedback on each trial. When the subjects saw the stimulus in a trial, they had to indicate their own estimate on the reward probability of the stimulus. After indicating probability estimate, she or he would receive a feedback on whether they won a reward or not.

Let $s_{1},s_{2}$ denote the two stimuli subjects faced in a context. In all the models we fit, we modeled the learning of $s_{1}$ and $s_{2}$ separately with different learning-rate parameter $\alpha_{1}$ and $\alpha_{2}$.

**Uncertainty- and reference-dependent (URD) model**

In trial t, after receiving the reward outcome (1=reward, 0=no reward), the average reward probability estimate $\hat{P}_{avg}$ is updated according to the delta-learning rule

$\hat{P}_{avg}\left(t+1\right)=P_{avg}\left(t\right)+\alpha_{avg}\delta_{avg}\left(t\right)$ (3)

where

$\delta_{avg}\left(t\right)=R(t)-\hat{P}_{avg}(t)$. (4)

Subjects then compute stimulus reward probability $s_{i}$ --$\hat{P}_{S_{i}}\left(t\right)$-- using the following context-dependent computation and use it to indicate his or her probability estimate

$\hat{P}_{S_{i}}\left(t\right)=\hat{P}_{S_{i}}\left(t\right)+\sigma_{S_{i}}\left(t|n_{past}\right)\left[\hat{P}_{S_{i}}\left(t\right)-\hat{P}_{avg}\left(t+1\right)\right]$ (5)

where $\sigma_{S_{i}}$ is the standard deviation of past reward outcomes associated with $s_{i}$ in the last $n_{past}$ trials and $\hat{P}_{avg}$ is the average reward probability of the context. $\hat{P}_{S_{i}}$ is then updated according to the delta-learning rule

$\hat{P}_{S_{i}}\left(t+1\right)=\hat{P}_{S_{i}}\left(t\right)+\alpha_{S_{i}}\delta_{S_{i}}\left(t\right)$ (6)

where

$\delta_{S_{i}}(t)=R(t)-\hat{P}_{S_{i}}(t)$. (7)

Here we consider the learning of average reward probability of a context – similar to Palminteri et al. (2015; also cited in the main text). Note: Here average reward probability is updated before stimulus reward probability. An alternative version is to update stimulus reward probability first before updating the average reward probability. These two versions in general produce similar fitting results, with the version outlined above fitting slightly better.

**Divisive normalization model (DN) (0-parameter, DN-1-0param)**

In this model, the context-dependent computation at the time of stimulus presentation is

$\hat{P}_{S_{i}}\left(t\right)=\frac{\hat{P}_{S_{i}}\left(t\right)}{\sum_{i=1}^{2} \hat{P}_{S_{i}}\left(t\right)}$ (8)

where $\hat{P}_{S_{i}}$ is then updated according to delta-learning rule in Eq. (4) and (5). Different from the previous model, this model does not require learning the average reward probability of the context $\hat{P}_{avg}$. In Eq. (8) there is no free parameter in the normalization computation and hence this model is referred to as the 0-parameter divisive normalization model.

**Divisive normalization model (DN) (1-parameter, DN-1-1param)**

In this model, we add a free parameter *a*

$\hat{P}_{S_{i}}\left(t\right)=\frac{\hat{P}_{S_{i}}\left(t\right)}{a+\sum_{i=1}^{2} \hat{P}_{S_{i}}\left(t\right)}$ (9)

**Divisive normalization model (DN) (2-parameter form)**

In this model, there are two free parameters *a* and *b*

$\hat{P}_{S_{i}}\left(t\right)=\frac{b+\hat{P}_{S_{i}}\left(t\right)}{a+\sum_{i=1}^{2} \hat{P}_{S_{i}}\left(t\right)}$ (10)

**Divisive normalization model (DN) (2-parameter form) from** [1]

In this model, there are two free parameters *a* and *b*

$\hat{P}_{S_{i}}\left(t\right)=\frac{b\hat{P}_{S_{i}}\left(t\right)}{1+a\sum_{i=1}^{2} \hat{P}_{S_{i}}\left(t\right)}$ (11)

**Range normalization model (RN) (0-param form, RN-0param)**

In this model, the context-dependent computation at the time of stimulus presentation is

$\hat{P}_{S_{i}}\left(t\right)=\frac{\hat{P}_{S_{i}}\left(t\right)-\min\left(\hat{P}_{S_{1}}(t),\hat{P}_{S_{2}}(t)\right)}{\max\left(\hat{P}_{S_{1}}(t),\hat{P}_{S_{2}}(t)\right)-\min\left(\hat{P}_{S_{1}}(t),\hat{P}_{S_{2}}(t)\right)}$ (12)

where $\hat{P}_{S_{i}}$ is then updated according to delta-learning rule in Eq. (4) and (5). Different from the previous model, this model does not require learning the average reward probability of the context $\hat{P}_{avg}$. In Eq. (12) there is no free parameter in the normalization computation and hence this model is referred to as the 0-parameter range normalization model.

**Range normalization model (RN) (1-parameter form, RN-1param)**

In this model, there is one free parameter *a*

$\hat{P}_{S_{i}}\left(t\right)=\frac{\hat{P}_{S_{i}}\left(t\right)-\min\left(\hat{P}_{S_{1}}(t),\hat{P}_{S_{2}}(t)\right)}{a+\max\left(\hat{P}_{S_{1}}(t),\hat{P}_{S_{2}}(t)\right)-\min\left(\hat{P}_{S_{1}}(t),\hat{P}_{S_{2}}(t)\right)}$ (13)

**Range normalization model (RN) (2-parameter form)**

In this model, there are two free parameters *a* and *b*

$\hat{P}_{S_{i}}\left(t\right)=\frac{b+\hat{P}_{S_{i}}\left(t\right)-\min\left(\hat{P}_{S_{1}}(t),\hat{P}_{S_{2}}(t)\right)}{a+\max\left(\hat{P}_{S_{1}}(t),\hat{P}_{S_{2}}(t)\right)-\min\left(\hat{P}_{S_{1}}(t),\hat{P}_{S_{2}}(t)\right)}$ (14)
