# Supplementary material for: Context effects on probability estimation
Source: PLoS Biol. 2020 Mar 5;18(3):e3000634. doi: 10.1371/journal.pbio.3000634 (PMC7077880; doi:10.1371/journal.pbio.3000634)
Supplement: S1 Table — Cluster-level inference was performed (familywise error corrected at p < 0.05) using Gaussian random field theory with a cluster-forming threshold p < 0.001 (z > 3.1). (DOCX) [file pbio.3000634.s009.docx]

**S1 Table**

|  | x | Y | z | cluster size (voxels) | z statistic |
| --- | --- | --- | --- | --- | --- |
| Visual cortex | -20 | -88 | -18 | 5861 | 6.33 |
| Superior Temporal Gyrus (left) | -58 | -10 | -2 | 661 | 4.35 |
| Precuneous cortex | -10 | -52 | 6 | 526 | 4.11 |
| Superior Temporal Gyrus (right) | 58 | -2 | -2 | 479 | 4.8 |
| Anterior cingulate cortex | 2 | 34 | 2 | 399 | 4.31 |
