# Supplementary material for: Context effects on probability estimation
Source: PLoS Biol. 2020 Mar 5;18(3):e3000634. doi: 10.1371/journal.pbio.3000634 (PMC7077880; doi:10.1371/journal.pbio.3000634)
Supplement: S2 Table — We performed nonparametric permutation test using the TFCE option in randomise (FSL) and performed 5,000 permutations. The p-value represents the familywise error corrected p-value. FSL, FMRIB software library; TFCE, threshold-free cluster enhancement. (DOCX) [file pbio.3000634.s010.docx]

**S2 Table**

|  | x | Y | z | cluster size (voxels) | p-value |
| --- | --- | --- | --- | --- | --- |
| Visual cortex | -20 | -88 | -16 | 24843 | 0.002 |
| Medial prefrontal cortex | -10 | 56 | 8 | 601 | 0.033 |
| Anterior cingulate cortex | 2 | 32 | 4 | 369 | 0.025 |
