# Supplementary material for: Context effects on probability estimation
Source: PLoS Biol. 2020 Mar 5;18(3):e3000634. doi: 10.1371/journal.pbio.3000634 (PMC7077880; doi:10.1371/journal.pbio.3000634)
Supplement: S3 Table — We performed nonparametric permutation test using the TFCE option in randomise (FSL) and performed 5,000 permutations. The p-value represents the familywise error corrected p-value. FSL, FMRIB software library; TFCE, threshold-free cluster enhancement. (DOCX) [file pbio.3000634.s011.docx]

**S3 Table**

|  | x | Y | z | cluster size (voxels) | p-value |
| --- | --- | --- | --- | --- | --- |
| Visual cortex | 28 | -86 | 22 | 2598 | 0.007 |
| Precuneous cortex (left) | -6 | -62 | 10 | 130 | 0.048 |
| Precuneous cortex (right) | 22 | -48 | 12 | 65 | 0.048 |
