# Supplementary material for: Context effects on probability estimation
Source: PLoS Biol. 2020 Mar 5;18(3):e3000634. doi: 10.1371/journal.pbio.3000634 (PMC7077880; doi:10.1371/journal.pbio.3000634)
Supplement: Attachment — Submitted filename: reply2reviewers_PLOSBio_final.docx [file pbio.3000634.s013.docx]

Dear Editors,

We thank the Editors for taking the time to review our manuscript, especially your suggestion on collecting additional data to strengthen the conclusion of the paper. We have both performed additional analyses and conducted 4 new behavioral experiments (N=80 subjects) to strengthen the conclusions. In particular, (1) we fit our data at the individual subjects’ level and fit the Rescorla-Wagner type learning models (Reviewers 1 and 2), (2) we addressed the relation between probability estimation and choice behavior by looking at whether the individual differences in the degree of context effect on probability estimate (all 3 Reviewers) correlate with individual differences in choice behavior, (3) we included IPS and anterior insula in multivoxel pattern analysis (Reviewer 3).

Please see our response in blue below.

Reviewer remarks:

Reviewer #1: This paper looks at context effects in probability learning. They find that, particularly for uncertain options, a context of higher probability (compared to lower) alternatives decreases the perceived probability (and vice versa). This is a relatively new area in neuroscience in which there has not been that much work. However, one notable study – Palminteri et al.  (2015) (‘contextual modulation of value signals in reward and punishment learning’) - is quite similar in key aspects:  the study also shows that probabilities are perceived differently depending on the context of the other alternatives being present and identified vmPFC and dmPFC/dACC as related to this phenomenon. What is conceptually new here is that the authors show that contextual effects are stronger for stimuli about which there is more uncertainty (though potentially an additional control experiment may be needed to rule out alternative explanations). Furthermore, the present study is very thoroughly done; in particular it is noteworthy that first, the behavioural results are replicated in a second sample. Second, results are shown both in a model-based and model-free framework, meaning that small arguments about how the models were specified do not affect the general value of the finding. Third, results are shown in terms of ratings as well as choices post-task, highlighting the robustness of the effect.

Thank you very much for your detailed and thoughtful comments, and for pointing out the thoroughness of the study.

Major comments:
Introduction/Discussion
1.      Palminteri et al. (2015) is only mentioned in passing. However, this study actually appears to be quite similar to the present one – both in terms of behavioural and neural effects - and that should be acknowledged and discussed

We thank the reviewer for pointing out the similarity between our study and that of Palminteri et al and we agree that our study adds a novel conceptual element in showing stronger contextual effects with more uncertainty. We would also like to point out that Palminteri et al study on value estimation which is an aspect of decision-making that is distinct from probability estimation which we have studied here. We have therefore amended the introduction (lines 111-113) and discussion (lines 1051-1071) to clarify the connection between our study and that of Palminteri et al.

Results - behaviour
2.      The authors highlight the role of uncertainty to explain the differences between the conditions where they find strongest effects (50%) and where they don’t (10% and 90%). However, it is also the case that the most certain options (i.e. 10% and 90%) are at the same time also those that are on the boundary of the rating scales. An alternative approach would be to use ‘noisy magnitudes’ (e.g. a stimulus is associated with reward drawn from a standard deviation with mean 50 points and with a standard deviation of 10 points). In this case, noisiness could be manipulated without approaching the boundary of the rating scale. If the authors want to reinforce the point about uncertainty, I would recommend collecting an additional behavioural sample with this manipulation of the task.

We agree with the concern that the more certain options are near the boundary of the rating scales and have therefore conducted three new behavioral experiments to address this point. The results of all of these new experiments support our model of interaction between uncertainty and reference-dependent computation. We thank the reviewer for your interesting suggestion of using a noisy reward magnitude manipulation. However our study’s focus is on probability rather than value estimation, so we added experiments that directly manipulated probability while still addressing the concern. Specifically, we added an experiment in which we studied probabilities farther from the boundary of the rating scale ([30%, 50%, 70%], n=20 subjects, Experiment 5, Lines 621-640). We also ran two sets of conditions in which we examined probability estimation solely at one end ([10%, 30%, 50%], 20 subjects, Experiment 3, lines 561-586) or the other [30%,50%,70%], 20 subjects, Experiment 4, Lines 588-619) of the probability scale, also allowing us to disassociate the boundary of the rating scale and the uncertainty. We also have added discussion about the issues of the rating scale and how these results support our conclusion of the interaction of uncertainty and reference-dependent computation (Lines 642-660).

3.      Modelling: the authors use as comparison model one adapted from Louie et al. (divisive normalization). I have several questions about this:
a.      Why does this model show an asymmetry in what it predicts for the 10% and 90% stimuli?

Thank you for allowing us to clarify. In the simplest form of divisive normalization – Ps=fs/(fs+fOS) – the normalized value for 10% in the [10%,50%] context is 0.1/(0.1+0.5)=0.167 and in the [10%,90%] context is 0.1/(0.1+0.9)=0.1. The difference is 0.067, a relatively small difference. By contrast, for 90%, the normalized value under [10%,90%] is 0.9/(0.1+0.9)=0.9 and under [50%,90%] is 0.9/(0.5+0.9)=0.643. The difference is 0.257, a much bigger difference than the one observed in 10%.

To make this point clear in the paper, we now include the above description in the main text (Lines 364-370).

b.      It seems that the authors have adapted the equation – i.e. they use Ps=(a+fs)/(b+fs+fOS), rather than in e.g. Khaw et al. (2017) P=a*fs/(1+b*fOverall). Why?

We were not aware of the Khaw et al. paper. Thank you for this reference. In our revision, we also fitted the Khaw et al. form. This form fits the data similarly as other normalization model (Fig. 7 and 8) (Lines 373-422). The Khaw form is referred to as DN-2-gamma. We note that at the request of another reviewer, we changed the acronyms of the models so that our model is now URD (Uncertainty and reference-dependent), divisive normalization is now DN, and range normalization is RN.

c.      I could not quite figure this out, but it might be relevant to consider literature on the ‘base rate’ phenomenon in a Bayesian framework (e.g. see Koehler (1996)). I have wondered whether participants somehow might think that an outcome is the result of a stimulus and the base rate of outcomes in that context. However, this is only a suggestion and authors can ignore this.

Thank you for this interesting suggestion. We view base rate and reference-dependency as conceptually distinct in terms of their prediction on context effect. We have included a short description of the relationship between our model and base-rate literature (Lines 709-714).

4.      Modelling: it appears from the methods (section Model fitting and model comparison> Maximum-likelihood estimation) that instead of fitting each person’s data, the model was fit on the average across participants. This is highly unusual and there is some data to suggest that this approach does not capture the real behaviour that well (Ahn et al., 2013). I would suggest that the authors either fit each person separately or that they use a hierarchical framework (e.g. can be done in the free software ‘Stan’, see Carpenter et al. (2017). These models should then be also used for model comparison.

Thank you for this suggestion. We now fit data at individual-subject level fitting using the original models and in addition implemented Rescorla-Wagner (R-W) model framework to these models (Lines 393-422). We found that our model did not significantly fit the data better than normalization models. However, qualitatively our model tends to capture the data better (lines 381-383, Fig. 7BC for group-data fit, Fig. 8AB for individual-data fit). We also note that with R-W framework the qualitative difference was gone. We discuss this on lines 398-409. In general, we find that our conclusions on reference- and uncertainty-dependency do not qualitatively depend on how we performed these analyses.

5.      Modelling: why do you use fs/foverall as a non-weighted sum of past trials, rather than the more typical Rescoral-Wagner weighted effect? Can you show by replacing e.g. fs in the regression by the past trial outcomes that in fact they are weighted equally?

We fit the R-W version of the models and the fits were better than non-weighted sum of past trials (Lines 393-422).

6.      Modelling: referring to table 4 (list of free parameters in models), it appears that the divisive normalization models did not allow for gamma (as in equation 6), i.e. over/underweighting of probabilities. By looking at figure 6, it appears that the uncertainty models particularly provide a better model fit when this gamma (or ‘wp’ in figure 6) is included. What happens if it is also included in the range normalization or divisize normalization models, are these then in fact the best performing models? I am asking this as it appears that in terms of BIC (so already correcting for the number of free parameters quite stringently), the range normalization/divisive normalization models are very similar to the uncertainty weighted models that don’t include gamma either.

Yes, after adding weighting function to fit normalization models we did find that BIC was not different between our model and normalization models. However, in contrast to our model, the normalization model still did not capture the no-effect on probability estimate at 90% (Fig. 7C). Hence, even though quantitatively we cannot no longer say our model is better, qualitatively it did perform a better job in characterizing context effect and lack thereof on probability estimate (Lines 373-391).

7.      Simulations: please include simulations in the supplement that show how well individual differences in behaviour can be recovered from data (see e.g. Melinscak and Bach (2019)). Also show to what extend the behavioural measure delta P (figure 7) correlates with the model parameters. As an aside please also explain why this is used instead of the model parameter? I assume it was done because the model was not fitted to each person individually?

Thank you for this suggestion. In the revision, we fit individual subjects’ data and simulate subjects’ choice based on the fits. We now show both fits and model simulations in the main text (Lines 393-422 for individual fits; Lines 424-427 for model simulations).

Delta P is a direct measure of context effect we got from the subjects’ probability estimate. Model parameters by themselves would not capture context effect. In our model, gamma – the parameter for probability weighting function – captures probability distortion, and sigma captures estimation noise. Rather, it is the context-dependent computations prescribed by the model with the estimated parameters that describe context effect (delta P).

8.      Data in figure 2: is the average made over the whole task, or only e.g. for the second half, when participants have already learnt? This appears relevant as if the effects get stronger with time (as they appear to do according to figure 4), a clearer picture for the 10% and 90% stimulus might emerge by only using later trials?

We have analyzed the second half of the experiment as suggested by the reviewer and found that there was no context effect both 10% and 90% probability estimates, while the context effect on 50% was significant. In addition, we also analyzed the first-half of the data and found the same result. In summary, regardless of analyzing the first half, second half or using all data, there was significant context effect on 50% but there was no context effect on 10% and 90% probability estimates (Lines 188-199).

FMRI:
9.      In figure 8 A-C it appears that there is no representation of the stimulus value in vmPFC. This seems pretty surprising given previous studies. Is it maybe because the regressor used to measure this was fs, rather than e.g. reward probability from a Rescorla-Wagner type model or the participant’s subsequent rating, which should maybe give the clearest prediction of a participant’s current value estimate. If that was the case, as follow-up analyses could be done similar to figure 5 in Palminteri et al. (2015) that use model-comparison to look at the context effects

Thank you for this suggestion. We have tried the reviewer’s suggestions – either using R-W model’s predicted probability estimate or simply using subjects’ probability estimate. In both cases, we did not find vmPFC to represent these variables at the whole-brain level. We then performed ROI analysis on VMPFC and ventral striatum using masks from Clithero and Rangel (2013) meta-analysis paper on the beta of probability estimate but still did not find the results to be significant (Fig. S7 in the supplement).

10.     Figure 8 – are only ‘positive effects’/ activations considered? E.g. commonly dACC is found to activate with inverse value at time of decision.

Thank you for this comment. We also considered negative effects of contrasts shown in Figure 8 but they were all not significant at the whole-brain level except for f_overall. However, after following another reviewer’s suggestion to perform more stringent cluster-level inference (Eklund et al., 2016; with cluster-forming threshold set at p<0.001 instead of p<0.01), the negative effect of f_overall was no longer significant. Accordingly, we updated this result in the paper (Lines 518-548) and presented tables of activations in the supplement (Tables S1-S4).

Minor comments:
-       Figure 8, it would be helpful if for each contrasts (lacking e.g. for D) it was stated explicitly where this was time locked to (rating or outcome).

Thank you for pointing this out. We have remade the figure the reviewer pointed to – now Figure 10 – by clarifying where each result is time-locked to. We also updated the figure based on the thresholding choice suggested by another reviewer (following Ekilund et al., 2016) (lines Lines 518-548).

-       Modeling: one feature of the proposed model I am not quite sure about: for a given trial, the model computes the probability estimate (PS) based on fs , foverall with a weight tau. And in a Rescorla Wagner framework, one would think that this is then stored for future computation. However, it seems that in the framework proposed here, series of outcomes are stored accurately, but then only skewed when one retrieves them to make a decision. If that is the assumption, it would be nice to discuss the rationale for this more.

We have fitted the R-W versions of our models and the fits tended to be better than models based on non-weighted past outcomes (Lines 393-422).

-       Figure 2, the figure would be easier to read if in A the y-axis was probability

We still feel including interval estimate (1~8) is important because it reflects the nature of the interval scale subjects used. To make it more readable, we added in the Figure legend the probability interval each value corresponds to (Lines 176-186).

-       FMRI: The authors find no difference between the contrasts for the stimuli in the different contexts. One complementary analysis would be analogous to Palminteri et al. where they first identified ROIs based on coding probability (see comments above) and then test in these with model-comparison whether probabilities derived from either model better explain the neural activity

Thank you very much for this suggestion. As we did not find evidence for either probability estimate or model-predicted probability estimate in VMPFC and ventral striatum in ROI analysis (see our response in Major comments 9.), we did not perform model comparison as in Palminteri et al.

References
Ahn W-Y, Krawitz A, Kim W, Busemeyer JR, Brown JW (2013) A model-based fMRI analysis with hierarchical Bayesian parameter estimation.
Carpenter B, Gelman A, Hoffman MD, Lee D, Goodrich B, Betancourt M, Brubaker M, Guo J, Li P, Riddell A (2017) Stan : A Probabilistic Programming Language. J Stat Softw 76 Available at: [https://www.osti.gov/pages/biblio/1430202-stan-probabilistic-programming-language](https://www.osti.gov/pages/biblio/1430202-stan-probabilistic-programming-language) [Accessed July 29, 2019].
Khaw MW, Glimcher PW, Louie K (2017) Normalized value coding explains dynamic adaptation in the human valuation process. Proc Natl Acad Sci 114:12696–12701.
Koehler JJ (1996) The base rate fallacy reconsidered: Descriptive, normative, and methodological challenges. Behav Brain Sci 19:1–17.
Melinscak F, Bach D (2019) Computational optimization of associative learning experiments. Available at: [https://osf.io/cgpmh/](https://osf.io/cgpmh/) [Accessed August 16, 2019].
Palminteri S, Khamassi M, Joffily M, Coricelli G (2015) Contextual modulation of value signals in reward and punishment learning. Nat Commun 6:8096.

Thank you very much for these references. We added Khaw et al., Koehler, and Palminteri et al. in our citations.


Reviewer #2: This is a very interesting paper using mathematical modeling combined with multivariate fMRI to study context effects in probability estimation.  As the authors note, there is a growing, but controversial, literature on context effects in valuation, but this paper tackles the other half of the decision-making problem by focusing on probabilities.  The authors use a simple but elegant design to present participants with three probabilities (10%, 50%, and 90%) in two different contexts (paired with the other two probabilities).  They demonstrate that these pairings produce repulsion effects, in particular for 50%, where 50% is judged as being higher probability when it is paired with 10% vs. 90%.  Importantly, the authors go on to show that this distortion in probability estimates carries over to a later binary choice task, biasing participants incentivized choices.  As an added bonus, the authors replicate these effects in a second replication experiment.  The behavioral effects are striking and an important finding on their own.  The authors go on to argue that these effects are best explained by a simple reference-point model, and that they cannot be explained by normalization models from the context-effects-in-valuation literature.  Finally, the authors show that the dACC correlates with these behavioral distortions, both at stimulus presentation and at feedback, while the vmpfc does so only at feedback.  Also, the temporal cortex tracks the stimulus probability, while the ventral striatum and fusiform track the overall probability within a context; these variables appear to be important for generating the overall probability estimations generated by the participants. 

I think that these are important, interesting, and novel findings.  To rigorously test the authors’ proposed model, I would have liked to have seen an experimental design with more than 3 probabilities and 2 levels of uncertainty, but I think that this can be left to future work. I don’t see the precise formulation or validation of the model as a critical element of the paper.  The paper puts forth a sensible model and shows that it outperforms other existing (or related) models.  If the authors can clarify a few things about their analyses and results, I would be happy to endorse publication. 

Thank you for the supportive comments about our manuscript and the suggestion to run more probability condition. We added three additional behavioral experiments looking at new combinations of reward probabilities: [30%, 50%, 70%] (Experiment 5), [10%, 30%, 50%] (Experiment 3), [30%,50%,70%] (Experiment 4). We have also performed an incentive-compatible experiment using the original probabilities [10%,50%,90%]. The results are reported on Lines 550-679 in the revised manuscript. These new findings are consistent with the original findings in that context effect on probability estimation is driven by the interaction between uncertainty and reference-dependent computations.

Introduction:
1. Page 6:  I don’t really understand this “second hypothesis” the way it is written.  Please revise. In particular, what does this mean: “potential context effects in probability estimation that are unique to valuation and thus points to neural systems dissociable from valuation.”?

Sorry for the lack of clarity. We have rewritten this part (Lines 97-106).

2. Why is there no mention of striatum in the introduction, especially since it plays an important role in the results?

Thank you. Yes, we now include striatum. Please see below for our modified text (Lines 90-91).

3. What does tracking recent and distant reward history have to do with probability, in terms of justifying looking at the dACC?

We agree with the reviewer that comparing recent and distant reward is not important here and rewrote this part (Lines 102-106).

Results:
4. For 10% and 90% what are the fractions of participants who show the expected effect (as you show with 50%)? It looks like there might be a couple of substantial outliers here.

For 10%, there are 18 subjects (out of 34) that showed the expected effect: these subjects gave smaller probability estimate of 10% in the [10%,90%] context than in the [10%,50%] context. For 90%, 23 subjects showed the expected effect: these subjects gave larger probability estimate of 90% in the [10%,90%] context than in the [50%,90%] context. We have included this information in the main text (Lines 195-199):

5. For Figure 3, t-tests seem inappropriate since these choice probabilities are not normally distributed.  I might suggest a non-parametric test like Mann-Whitney. (Essential)

Thank you for pointing this out. We now use bootstrapping – nonparametric resampling of data – to estimate the confidence interval of choice probability (Lines 1246-1251 in Methods to describe the bootstrap procedure) and use it to test whether the effect is significant. The results remained the same (Lines 218-224).

6. It seems important to show that participants’ behavior in the fMRI task correlates with their choice bias in the post-fMRI task.  (Essential)

Yes, they are related. We now report on Lines 227-246 and Fig. 3BCD.

7. In Fig. 4B it looks like the 50% paired with 10% was in fact better early on during pre-fMRI than the one paired with 90%.  Could the authors sub-sample their participants in such a way that this is not the case, and then still show the behavioral effect?  The control experiment helps, but it would be nice to unambiguously see the effect in both experiments.

Yes, they do show the effect as expected. There were 14 subjects who fit this criterion and yet they still showed the same context effect on probability estimate. We have put the results in the supplement (Fig. S2).

8. What did you expect to see in the response times?  Without a clear hypothesis, these results seem better suited to the supplements.

We did not have a specific hypothesis for the response time but had included them for completeness and have therefore now moved them to the supplement (Fig. S1).

9. Control experiment: Why so few participants? Given that the effects at 90% and 10% were previously borderline, I would have expected a slightly larger experiment here, especially given that it is purely behavioral.  Also, where are the statistics for the probability estimates? (Essential)

We have since conducted 4 new behavioral experiments in addition to the two experiments (fMRI and control, Experiments 1 and 2 respectively). Each of these new experiments had 20 subjects. In one of them (Experiment 6), we used exactly the same setup as the control experiment in the original submission except that subjects were given monetary incentive for being accurate in their probability estimate. We replicated the context effect on 50% and found no effect on 10% and 90% (Lines 661-678).

For the statistical test on probability estimate, we now use bootstrapping to compute the confidence interval for probability estimate and use it to statistically test context effect (Lines 159-161; Lines 1246-1251 in Methods to describe the bootstrap procedure).

10. Doesn’t the model make an even stronger prediction, which is that the coefficient on f_s should be the coefficient on -f_overall, plus one?  (This actually appears to be roughly true in Fig. 6B). Can the authors formally test this? And shouldn’t the authors be testing that tau is indeed close to sigma?  Does the model fit improve with a flexible tau?

Yes, that is what the model predicts. And yes, that is what we found after testing it. Thank you for this suggestion. We now test this separately for each probability. We added this result on Lines 335-339. We also explicitly tested tau as sigma in our model fitting. All the models we fit we used sigma (standard deviation of past outcomes) or sigma square (variance of past outcomes) when computing probability estimate.

11. Fig. 6C caption - there is a repeat here of UncRef-var-wp.  Also, I would suggest trying to come up with better names or acronyms for these models, rather than these awkward labels.

Thank you. We changed the acronym to URD for Uncertainty- and Reference-Dependent model throughout the paper. Also change divisive normalization to DN and range normalization to RN.

12. I’m not sure that I would bother including the normalization fits in the main text.  Without more background, these won’t make sense to most readers.  Perhaps it could just be a discussion point instead and you could point to results in the supplements?  I leave this up to the authors though.

Thank you for this suggestion. We feel that we should still include them because another reviewer had several comments even though most are for clarification purpose.

13. Figure 7 - something needs to be noted here regarding “voodoo correlations”.  While these correlations are significant, their magnitude is certainly inflated due to the fact you are looking at the peak correlations from many many tests.  One potential way to get around this issue, would be to identify the best voxels using one measure (delta_p) and then only use those voxels to rerun the analysis using the other behavioral measure (i.e. the choice bias in the post-fMRI task).  Or vice-versa. (Essential)

Thank you for this comment. We apologize for not being clear, but we are not looking at peak correlations from many tests. Instead, we use permutations to construct null distribution on correlation for the purpose of testing whether the actual correlation we obtained –that is, the correlation between delta_p and predicted delta_p based on multivoxel pattern — is significant. Even though the number of possible permutations is finite, it is very large and hence computationally expensive. To alleviate the cost, we adopted the same strategy as in Schmack et al. (2016) in that, for a given searchlight centered on a voxel, if the “peak” of the permuted correlations exceeds the actual correlation after #n permutations (#n is determined based on number of voxels in the ROI), we would label the searchlight centered on this voxel non-significant.

14. On p.23 you repeat the same results twice.  You describe the correlations with f_s and f_overall, then do so again. 

Thank you for pointing this out. We have corrected this error. Please see below. However, the other reviewer suggested that we follow the recommendation in Eklund et al. (2016) – a p<0.001 cluster-forming threshold when performing cluster-level inference. As a result, we no longer found significant stimulus reward frequency representation in superior temporal cortex. Hence, we updated our results (lines 439-446).

15. The results on reward prediction errors seem to come out of nowhere.  It would be useful to briefly explain why you did these analyses.  I assume that it was to show that you had enough power to detect probability effects, if they were there?

Yes, we want to show that with this dataset we were able to replicate the classical result on RPE and also on reward magnitude in VMPFC and striatum. So we should have enough power to detect probability effect if there were there. We added on Lines 518-548 saying that we replicated classical results on RPE and reward magnitude.

16. Did you test if reward prediction errors were better explained with vs. without the observed context effects?

No we did not. The reason is it would be very difficult to test this due to issue on collinearity in GLM. To directly test this, we would have to include both the prediction error provided by the context model and prediction error without considering context effect in the same GLM. However, given that context effect is seen primarily in 50%, but not in 10% and 90%, these two regressors will be highly correlated. In fact, we found that the overall correlation between these two is over 0.9 and therefore it is not reasonable to include both in the same GLM. Based on this, we would also expect that, in the case of implementing each separately in different GLMs, the ROIs such as ventral striatum would have identical beta of prediction error between the context and absolute models.

Discussion:
17. If f_s is really represented in temporal cortex activity, why don’t participants just report that? Why combine it with f_overall? In other words, if there is an unbiased measure of the stimulus probability, why aren't participants reporting it?

After applying the new cluster-forming threshold suggested by another reviewer (p<0.001 recommended in Eklund et al., 2016), we no longer found temporal cortex to represent f_s. We can no longer claim that there is a significant representation of f_s. Therefore, we toned down the statement (Lines 796-807).

Methods:
18. What does it mean that “the order of [the visual stimuli] presentation was randomized for each block separately”?  Were the blocks randomly ordered or not? It seems they were in the fMRI session, but what about pre-fMRI? (Essential)

Yes, the blocks were randomly ordered in both pre-fMRI and fMRI sessions. We now clarify it (Lines 935-936). What we meant here is that since there were two stimuli in each block, the order in which they appeared within a block was randomized. We clarify this in Methods by saying that “The order of the two stimuli presented within each block was randomized.” (Lines 837-938, Lines 943-944).

19. Was there any incentivization for the guessing task?

No, there was no incentive for the probability estimation task. We clarified it (Lines 910-913). We performed a new experiment that provided incentive to estimate reward probability and replicated the original results (Results: Lines 661-679; Methods: Lines 997-1005).

20. In Model 1 why interact PE with MAG? How did you define PE?

Here PE is the actual outcome (1 or 0) minus the probability estimate. Because we jittered the reward magnitude (1 to 5 dollars randomly), we also included the interaction term – PE x MAG – in the GLM.

21. Is there a citation for this particular type of permutation test, or did you come up with it yourselves? (Essential)

This is based on Schmack et al. (2016; Cereb Cortex: Predicting subjective affective salience from cortical responses to invisible object stimuli). We added on Lines 484-485 when mentioning permutation test.

22. It seems arbitrary to use the last 10 trials to model f and sigma.  Why this measure and not the true values, the observed values, or some RL-like version?

We did it for convenience. It is arbitrary to select 10 and not ideal. In general, we found that the model fits – in BIC values – decreased as a function of window lengths and would vary little after 5 trials into the past. We clarified this (Lines 1184-1186) and showed the results are shown in the supplement (Fig. S3). We also fitted RL-like version and found that our model did not do significantly better than normalization models (Lines 393-422). However, qualitatively our model tends to describe the data better (Lines 380-391, and Fig. 7BC for results on fitting group data).

Reviewer #3: This interesting manuscript describes an fMRI study testing whether probability estimations and related choices are affected by context, in comparable ways as valuation processes are. In 2 behavioral experiments, the authors show that participants’ probability estimates for a given series of cue-related reward outcomes were indeed systematically biased by the overall probability of reward (manipulated by differing reward probabilities for a second cue presented in the same experimental block). This bias was strongest for intermediate probabilities (50%) and appeared well captured by a new computational model that the authors systematically test against other competing models derived from the valuation literature. Analysis of the fMRI data acquired during one of the experiments showed that the context effects were reflected in multi-voxel activity patterns in the ACC and vmPFC, and that the strength of these neural context effects correlated across participants
with the strength of the behavioral context effects. The authors conclude that the vmPFC may constitute a shared neural substrate underlying both probability estimation and valuation and that context may affect both these processes.

I enjoyed reading this manuscript: It addresses an interesting and relevant question with an innovative experimental approach; the research methods are sophisticated and generally applied in a sound way; the results are reasonably clear-cut; and the manuscript is well-written and provides interesting discussion. However, there are several issues with the motivation, experimental design, procedures, and results that the authors should address to render their manuscript more conclusive and balanced.

Thank you for your supportive, thorough and constructive comments.

(1)     The authors focus in their ROI analyses on the OFC/vMPFC and ACC, based on the previous literature. However, it is not clear to the reader whether these are really the only two areas that could be expected to be involved in probability coding, based on a truly a-priori review of the literature on probability coding. Off the top of my head, I would have expected also the anterior insula and the IPS to be consistently found in such studies, but there may be even more areas. To prevent the impression of “post-hoc” hypotheses, and to counteract possible bias, the authors should go over pertinent meta-analyses and test all the ROIs suggested by the prior literature to be involved in probability coding. Reporting these analyses is valuable even if they yield null results. 

We surveyed meta-analysis papers on probability coding and found one earlier paper (Mohr, Biele, Heekeren, 2010, JN). We reached out to the authors for masks but never heard from them. We also contacted senior author from Bartra et al. (2013, meta-analysis paper on subjective value) because it was pointed out in their Discussion that meta-analysis papers on risk/probability coding was needed and the response we got was that he was also only aware of the Mohr paper. Finally, we went to Neurosynth and extracted two masks, one using probability as keyword and the other using uncertainty as keyword. These two masks showed extensive overlap and include IPS and anterior insula, in addition to mPFC and dACC. Therefore, we use the conjunction of these two maps for our ROI analyses. Among the newly tested ROIs, only the right IPS showed significant results. We have updated the results (Lines 458-516, and Fig. 9) and the Introduction and Discussion to account for the changes.

(2)     The authors find that context effects are strongest for 50% probability, compared to 10% and 90%. The formal model suggests that this differential effect reflects different levels of outcome uncertainty. However, a convincing test of this quantitative hypothesis would require many more probabilities than just the somewhat special cases of 50% and two values at the very extreme ends of the scale (10% and 90%): We know that the latter two probabilities are perceived in distorted ways; moreover, there are ceiling and floor effects that may affect estimations. I do like the new model proposed by the authors and I appreciate the careful model comparisons, but the dataset is not rich enough to fully test the quantitative predictions afforded by the model. Ideally, the authors would conduct a control experiment that also includes intermediate probabilities (say 30% and 70%) for which no strong perceptual distortions have been reported but that nevertheless differ in outcome uncertainty from the special case of 50%. At the very least, the authors should discuss this caveat to not give the impression that all predictions of their interesting new model have been tested by the somewhat restricted experimental design.   

Thank you very much for this suggestion. We agree that additional experiments on probability estimation need to be done and therefore ran 4 new behavioral experiments that examine context effect at different probabilities and contexts (Lines 550-679).

Specifically, we added an experiment in which we studied probabilities suggested by the reviewer ([30%, 50%, 70%], n=20 subjects, Experiment 5). We also ran two sets of conditions in which we examined probability estimation solely at one end ([10%, 30%, 50%], 20 subjects, Experiment 3) or the other [30%,50%,70%], 20 subjects, Experiment 4) of the probability scale. Finally, we tested whether incentive compatibility changes the original findings (Experiment 6). Together, these results support our conclusion on the interaction of uncertainty and reference-dependent computation.

(3)     In the fMRI experiment, the authors chose to have a category boundary at 50%, meaning that a perceived probability of 50% forces subjects to choose whether to opt for the higher or the lower response category. The authors show in a behavioral control experiment that this probably did not cause their behavioral effects, but the neuroimaging data may still be affected by it. This is particularly worrisome as the ACC is known to be involved in situations with response conflicts such as those present in the scenario described above (one probability being associated with two competing responses). The authors should discuss this caveat, perhaps focusing on why specifics of the activity patterns they observe (anatomical location, involvement in different stages of the design) support the view that response conflict is less likely to explain their results than their alternative proposal.

Thank you for bringing this interesting literature up as a potential interpretation of our dACC finding. We have since added a paragraph in Discussion and provide two pieces of evidence that conflict with this interpretation (Lines 808-827).

(4)     The authors constructed different sequences of reward events by random draws from distributions with the desired mean probability. This strategy is good in controlling for many things; however, it invariably means that different participants saw different sequences and were exposed to actual frequencies that differed from the desired target frequency. The authors account for this property of their design in the model they build and in some of their analyses, but not in all of them. For instance, for the analyses presented in Figure 2, were the individual probability estimates correlated with the actual frequencies experienced by each individual? Likewise, how were the effects in presented in Figure 3 correlated with individual frequencies? The authors should present these analyses to convince the reader that their model accurately explains each individual’s frequency perceptions, not just the average tendency in the pooled data.

Thank you. Yes, the individual subjects’ probability estimates correlated with actual frequency experienced by each individual (Lines 200-205) and also correlated with their choice behavior (Lines 226-234).

(5)     The authors state that the ventral striatum (VS) codes outcome magnitude and refer to Table 2, but this table does not contain the VS?

Thank you for pointing this out. VS was part of the cluster that includes the mPFC. However, after applying p<0.001 as the cluster forming threshold, as suggested by the reviewer (following Eklund et al., 2016), we no longer see the VS result. The mPFC result still holds (Lines 518-548). Please also see Figure 10 in the main text.

(6)     The authors fit the model with a moving window extending over the last 10 trials. How did they select this number? Are their results robust to different window lengths?

We did it for convenience. It is arbitrary to select 10 and not ideal. In general, we found that the model fits – in BIC values – decreased as a function of window lengths and would vary little after 5 trials into the past. We clarified this (Lines 1184-1186) and showed the results are shown in the supplement (Fig. S3).

(7)     On page 9, the authors write: “We found significant context effect on probability estimate when reward probability was 50%, but not at 10% (t=1.9419, df=33, p=0.0607) or 90% (t=1.9441, df=33, p=0.0605).“ Are these statistics for the analyses of the data acquired for these two probabilities? In that case, I do not think that p-values of p=0.06 provide convincing evidence that there is no context effect. The authors should show that the effects are significantly stronger for 50% than for the two other probabilities, in direct statistical comparisons of these effects.

Thank you for this important comment. We have since compared the size of the context effect between different probabilities and found that the size was significantly bigger in 50% than 10% or 90% (Lines 188-193).

(8)     The authors present the context effects on neural probability representations (in dACC and vmPFC) and the neural representation of contex (in VS and temporal areas) in isolation, even though these representations should be linked if their model and interpretation is correct. Please report whether the strength of these two sets of effects indeed correlates across subjects as would be expected. The authors may also consider conducting connectivity analyses to analyse how the information is shared between these two sets of regions (but such new analyses are clearly optional and not required for their results to stand).

Thank you for this great suggestion. However, it would be hard to evaluate the first suggestion – correlating the effect of context-dependent probability representations (dACC and vmPFC) with representations of reward frequency information (VS and temporal areas) – because context-dependent probability representations (dACC, vmPFC) came from between-subject multivoxel pattern analysis, which does not give us an estimate on the strength of the effect on probability at the individual subject’s level.

We did follow the reviewer’s second suggestion, which was to perform connectivity analysis. We performed a PPI analysis using dACC as the seed region: a sphere ROI in dACC centered at (x2,y30,z18) – the voxel with the strongest effect in our multivoxel pattern analysis. We found significant decrease in functional connectivity between dACC and mPFC at the time of stimulus presentation. We did not find dACC to show significant changes in functional connectivity with ventral striatum (that represents overall frequency of reward) at both time windows. We put these results in the supplement (page 11).

(9)     The manuscript is unclear as to whether the authors used cluster- or voxel-level inference. I suspect the former given that they mention a cluster-forming threshold, but this should be stated explicitly. Moreover, the authors should ensure they properly account for the problems associated with some types of cluster-level inference as highlighted in Eklund et al 2016 PNAS.

We indeed performed cluster-level inference in our initial submission and used a more lenient cluster-forming threshold at p<0.01. We have since used p<0.001 as the cluster-forming threshold for cluster-level inference using Gaussian random field theory and non-parametric permutation test (both recommended in Eklund et al., 2016). We still found reward magnitude representation in mPFC at the whole-brain level and reward prediction error and overall frequency of reward in ventral striatum using ROI analysis. We clarified this (Lines 438-440) and updated these results in the revised paper (Lines 518-548).

(10)    On Page 33, the authors write: “It reflects that seeing a stimulus with intermediate probability of reward like 0.5 feels better when the other stimulus present in the context is much worse than 0.5 than when the other stimulus carries a much better chance of reward.” What is the evidence for these presumed feelings? The authors either need to present this or refrain from inferring on feelings based on fMRI data alone.

Thank you for pointing this out. We took the liberty of using the word “feelings” to describe better or worse than expected reward probability as the driving force behind the context effect we observed. Indeed, since we did not ask subjects to rate their feelings (we asked them to indicate probability estimate), we have no concrete evidence to say that the context effect reflects feelings. Hence we take this sentence out from the Discussion.
